# Numbers of People with HIV/AIDS Reported and Not Reported to Surveillance in Japan

**DOI:** 10.2188/jea.14.182

**Published:** 2005-03-18

**Authors:** Shuji Hashimoto, Miyuki Kawado, Yoshitaka Murakami, Seiichi Ichikawa, Hirokazu Kimura, Yosikazu Nakamura, Masahiro Kihara, Kazuo Fukutomi

**Affiliations:** 1 Department of Hygiene, Fujita Health University School of Medicine.; 2 Epidemiology and International Health Research Section, Environmental Health Sciences Division, National Institute for Environmental Studies.; 3 Nagoya City University School of Nursing.; 4 Department of Public Health, Yokohama City University School of Medicine.; 5 Department of Public Health, Jichi Medical School.; 6 Kyoto University School of Public Health.; 7 National Institute of Public Health.

**Keywords:** HIV, Acquired Immunodeficiency Syndrome, surveillance, trend, estimation

## Abstract

BACKGROUND: Trends in the numbers of Japanese patients with human immunodeficiency virus (HIV) and acquired immunodeficiency syndrome (AIDS) reported to the HIV/AIDS surveillance system in Japan were examined. We attempted to estimate the cumulative number of Japanese with HIV, including people with HIV not reported to the surveillance.

METHODS: Data from the HIV/AIDS surveillance in Japan up to the end of 2002 were available. The number of unreported HIV cases was estimated using the back-calculation method. To evaluate this method, the number of reported HIV cases up to 1996 (before highly active antiretroviral treatments were widely available in Japan) was compared with the number estimated by the same method.

RESULTS: The number of AIDS cases who were initially reported as having AIDS without having been reported as HIV-infected markedly increased as did the number of reported HIV cases. The number of AIDS cases who had been initially reported as HIV-infected and who were then reported as AIDS progression increased up to 1996 but decreased in the period of 1997-2002. The cumulative number of people with HIV at the end of 2002 was estimated as 14,000, which was 4.2 times higher than the number of reported HIV cases. The cumulative number of HIV cases reported up to 1996 was nearly equal to the number estimated by the above-mentioned method.

CONCLUSIONS: HIV infection would appear to be spreading widely among Japanese population. The number of HIV cases actually reported to surveillance might still be low.

HIV/AIDS surveillance, which reveals trends in the numbers of patients with human immunodeficiency virus (HIV) and acquired immunodeficiency syndrome (AIDS) for planning and evaluating countermeasures against HIV/AIDS, has been conducted in many developed countries including Japan.^1^^-^^3^ However, such trends based on surveillance data must be carefully interpreted.

The number of HIV cases reported to surveillance reliably represents the number of people diagnosed with HIV if the proportion of people diagnosed with HIV who reported to surveillance is sufficiently high. In recent years, people diagnosed with HIV can prevent or delay the progression to AIDS by undergoing highly active antiretroviral treatments, including combination regimens such as two nucleoside reverse transcriptase inhibitors plus one protease inhibitor.^4^^-^^6^ The trend in the number of AIDS cases who had been initially reported to surveillance as HIV-infected and were subsequently reported as having progressed to AIDS (secondarily reported AIDS cases) would reflect the effects of highly active antiretroviral treatments. Furthermore, the trend in the number of AIDS cases who were initially reported as having AIDS without having been previously reported as HIV-infected (initially reported AIDS cases) might reflect the number of people with undiagnosed HIV, which is important for monitoring through surveillance.^7^

In Japan, the proportion of those reported to surveillance among people diagnosed with HIV and AIDS was indicated to be sufficiently high.^8^ Highly active antiretroviral treatments have been widely used in cases diagnosed with HIV and/or AIDS since 1997.^9^ However, the trend in the number of non-Japanese with HIV and AIDS has been affected by arrivals to and departures from Japan.^10^

In this study, trends in the numbers of Japanese HIV and AIDS cases reported to surveillance in Japan up to the end of 2002 were examined. Using the surveillance data, we attempted to estimate the cumulative number of Japanese with HIV including those with HIV not reported to surveillance.

## METHODS

### HIV/AIDS surveillance in Japan

HIV/AIDS surveillance in Japan, organized by the Ministry of Health, Labour and Welfare of the Japanese government, was started in 1984.^3^^,^^11^ Both HIV infection and AIDS are notifiable conditions and are reported by the diagnosing physician (cases infected through blood products are not included). In the surveillance, two types of reporting forms are used; Form 1 is for the initial identification of HIV seropositivity or AIDS, and Form 2 is for cases identified as having progressed from being HIV positive to developing AIDS or from having AIDS to death. Form 1 includes sex, age, nationality, HIV/AIDS status, date of diagnosis and route of infection, while Form 2 includes all of those except for the route of infection.

### Trends in the numbers of HIV and AIDS cases reported to surveillance

The annual trends in the numbers of Japanese HIV cases reported to surveillance and AIDS cases initially reported up to the end of 2002 were examined using the data of Form 1. In addition, the annual trends in the numbers of secondarily reported AIDS cases were observed using the data of Form 2.

The numbers of secondarily reported AIDS cases up to 1996 and in 1997-2002 (when highly active antiretroviral treatments were widely available in Japan) were compared with their numbers expected under the condition that HIV cases received no active antiretroviral treatments. We assumed that under this condition, each reported HIV case had the expected cumulative probability of AIDS progression over a 20-year period previously reported: 0.00, 0.005, 0.03, 0.09, 0.15, 0.22, 0.29, 0.36, 0.43, 0.50, 0.54, 0.58, 0.62, 0.66, 0.70, 0.74, 0.78, 0.82, 0.86 and 0.90 at 1-20 years after HIV infection, respectively.^12^ Under the assumption, the expected number of AIDS cases progressed from reported HIV cases was calculated as the total of the expected cumulative probability of AIDS progression for such cases corresponding to the elapsed years after their report of HIV infection.

### Cumulative number of people with HIV estimated from surveillance data

The cumulative number of Japanese with HIV at the end of 2002 was estimated as the number of HIV cases reported to surveillance plus the estimated number not reported. The number of reported HIV cases was obtained from the surveillance data. The number of unreported HIV cases was estimated using the back-calculation method^13^ and the surveillance data as follows.

We assumed that unreported HIV cases received no active antiretroviral treatments, and that each unreported HIV case had the expected cumulative probability of AIDS progression over a 20-year period above-mentioned. We also assumed that the distribution of years after HIV infection among unreported HIV cases was equal to that among reported HIV cases. Under these assumptions, the mean expected cumulative probability of AIDS progression for unreported HIV cases was calculated as the mean of the expected cumulative probabilities of AIDS progression corresponding to the elapsed years after the report of HIV infection among reported HIV cases. The number of unreported HIV cases was estimated as the number of initially reported AIDS cases divided by this mean expected cumulative probability of AIDS progression.

To evaluate the method for estimating the number of unreported HIV cases, the cumulative number of HIV cases reported up to 1996 (before highly active antiretroviral treatments were widely available in Japan) was compared with the number estimated by the same method.

## RESULTS

### Trends in the numbers of HIV and AIDS cases reported to surveillance

**Figure 1.**
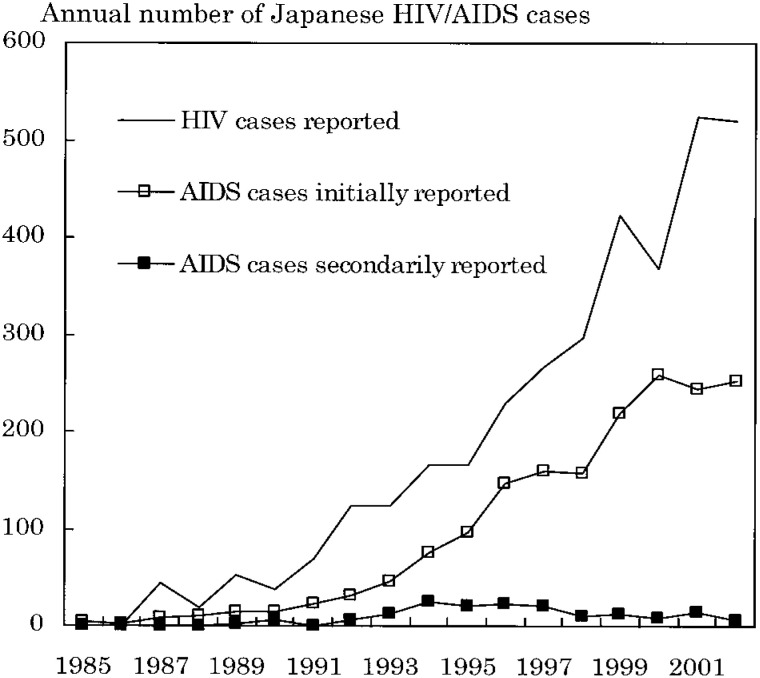
Annual trends in the numbers of Japanese HIV and AIDS cases reported to surveillance.

Figure 1 shows the annual trends in the numbers of Japanese HIV and AIDS cases reported. The number of initially reported AIDS cases markedly increased as well as the number of reported HIV cases. The number of secondarily reported AIDS cases increased up to 1996 and decreased thereafter (1997-2002).

**Figure 2.**
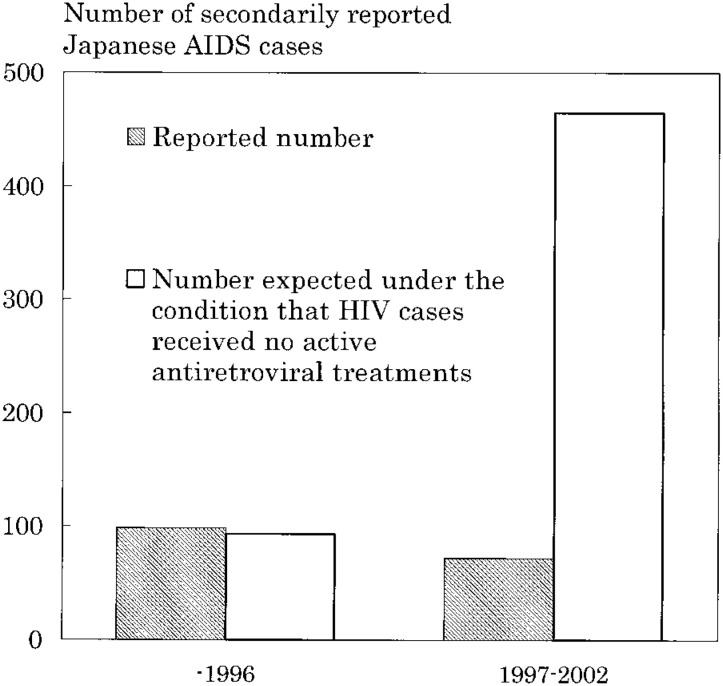
The number of Japanese AIDS cases secondarily reported to surveillance and its expected number.

Figure 2 shows the numbers of secondarily reported AIDS cases up to 1996 and in 1997-2002, and the numbers expected under the condition that HIV cases received no active antiretroviral treatments. The reported number was nearly equal to its expected number up to 1996, but was markedly lower than its expected number in 1997-2002 (i.e., the reported number of 72 vs. the expected number of 465).

### Cumulative number of people with HIV estimated from surveillance data

Table 1 shows the estimated cumulative numbers of Japanese with HIV and/or AIDS at the end of 2002. The mean of years after the report of HIV infection among reported HIV cases at the end of 2002 was 4.6 years. Under the assumptions that unreported HIV cases received no active antiretroviral treatments, and that the distribution of years after HIV infection among such cases was equal to that among reported HIV cases, the mean expected cumulative probability of AIDS progression among unreported HIV cases was calculated as 0.163. The number of unreported HIV cases was estimated as 11,000 (=the number of initially reported AIDS cases / the mean expected cumulative probability of AIDS progression among unreported HIV cases = 1,771/0.163). The cumulative number of people with HIV was estimated as 14,000 (=the number of reported HIV cases plus the estimated number of unreported HIV cases = 3,436 + 11,000), which was 4.2 times higher than the number of reported HIV cases.

**Table 1.** The estimated cumulative number of Japanese with HIV at the end of 2002.

	Progression to AIDS	Without progression to AIDS	Total
Reported as HIV- infected	171*	3,265*	3,436*
Unreported as HIV-infected	1,771*	9,000^†^	11,000^†^

Total	1,942*	12,000^†^	14,000^†^

* : the reported number

† : the estimated number

The cumulative number of HIV cases reported up to 1996 was 1,033, which was nearly equal to the 1,090 estimated by the above-mentioned method.

## DISCUSSION

The increase in the number of reported HIV cases indicated that the number of people diagnosed with HIV was increasing. The rise in the number of initially reported AIDS cases indicated that people with undiagnosed HIV were increasing. Thus HIV infection would appear to be spreading widely among the Japanese population. However, the increase up to 1996 together with the decrease in 1997-2002 in the number of secondarily reported AIDS cases suggested that progression to AIDS among many people diagnosed with HIV has been prevented or delayed due to the wide use of highly active antiretroviral treatments since 1997 in Japan.^9^ The number of persons with AIDS progression prevented in 1997-2002 might be evaluated by the number of secondarily reported AIDS cases in 1997-2002 compared with its number expected under the condition that reported HIV cases received no active antiretroviral treatments (the reported number of 72 vs. the expected number of 465). Further research is required.

The cumulative number of people with HIV was estimated as 14,000, which was 4.2 times higher than the number of reported HIV cases. These findings suggested that many people had HIV in Japan, that a large proportion of those not diagnosed had no opportunity to prevent or delay their progression to AIDS by undergoing highly active antiretroviral treatments, and that aggressive countermeasures must be taken to prevent HIV infection and provide opportunities to detect such potential HIV infection in Japan.

This study has several problems and limitations. The most critical problem involves the accuracy of the data from the HIV/AIDS surveillance system in Japan. Our results were affected by the breadth of the coverage and the possible duplication in reporting diagnosed HIV and AIDS cases.^3^^,^^14^ However, the proportion of people diagnosed with HIV and AIDS who reported to surveillance was seen to be sufficiently high.^8^ Although the secondarily reporting of AIDS cases was put on a voluntary basis after the Infectious Disease Control Law was enacted in April 1999 in Japan, no great decline in its coverage was suggested.^15^

In estimating the number of unreported HIV cases, we used the back-calculation method which has been widely employed for predicting the number of HIV and AIDS cases.^13^ In this method, the data on reported HIV cases and initially reported AIDS cases was used, whereas the data of secondarily reported AIDS cases was not. For applying other methods such as a system analysis, further data would be neccesary.^10^^,^^16^

The essential assumptions were that unreported HIV cases received no active antiretroviral treatments, and that the distribution of years after HIV infection for unreported HIV cases was equal to that for reported HIV cases. Using these assumptions, the mean expected cumulative probability of AIDS progression for unreported HIV cases was calculated. The former assumption would be reasonable because the proportion of people diagnosed with HIV reported to surveillance was found to be sufficiently high. Had the coverage of undiagnosed HIV cases reported to HIV/AIDS surveillance in Japan risen rapidly in recent years, the latter assumption would not be valid. There were no reports enabling us to reliably determine whether this assumption was valid or not in Japan.

Another assumption was that data on the expected cumulative probability of AIDS progression in the absence of active antiretroviral treatments previously reported were available.^12^ It would be safe to assume that HIV cases reported up to 1996 (before highly active antiretroviral treatments were widely available in Japan) would not have received active antiretroviral treatments, as was also true of unreported cases. The cumulative number of HIV cases reported up to 1996 was nearly equal to the number estimated by the same method under this assumption, suggesting that this assumption would be equally valid for HIV cases reported up to 1996 as well as for unreported cases.
